# The association between parental attitudes and alcohol consumption and adolescent alcohol consumption in Southern Ireland: a cross-sectional study

**DOI:** 10.1186/s12889-016-3504-0

**Published:** 2016-08-18

**Authors:** Eimear Murphy, Ian O’Sullivan, Derry O’Donovan, Ann Hope, Martin P. Davoren

**Affiliations:** 1 Department of Epidemiology and Public Health, University College Cork, 4th Floor Western Gateway Building, Western Road, Cork, Ireland; 2 Department of Public Health and Primary Care, School of Medicine, Trinity College Dublin, Dublin 2, Ireland

**Keywords:** Alcohol, Adolescent, Parent, Consumption

## Abstract

**Background:**

Alcohol plays a complex role in society. A recent study showed that over half of Irish adults drink hazardously. Adolescents report increased levels of alcohol consumption. Previous research has inferred the influence of the parent on their adolescent. Thus, the aim of the current study was to investigate the association between adolescent alcohol consumption and their parent’s consumption pattern and attitude toward alcohol use in Southern Ireland.

**Methods:**

A cross-sectional survey was undertaken in November 2014. This involved distributing a survey to adolescents (*n* = 982) in their final two years of second level education and at least one of their parents from a local electorate area in Southern Ireland. This survey included: alcohol use, self- reported height and weight, smoking status, mental health and well-being along with attitudinal questions. Chi-square tests and multivariate logistic regression were utilised.

**Results:**

A 37 % response rate was achieved. Over one-third (34.2 %) of adolescents and 47 % of parents surveyed reported hazardous drinking. Over 90 % of parents disagreed with allowing their adolescent to get drunk and rejected the idea that getting drunk is part of having fun as an adolescent. The majority (79.5 %) of parents surveyed believed that their alcohol consumption pattern set a good example for their adolescent. Multivariate logistic regression highlights the association between adolescent hazardous alcohol consumption and hazardous drinking by the father. Furthermore either parent permitting their adolescent to drink alcohol on special occasions was associated with hazardous alcohol consumption in the adolescent.

**Conclusion:**

The findings of this research notes a liberal attitude to alcohol and increased levels of consumption by the parent are linked to hazardous adolescent drinking behaviour. Future action plans aimed at combatting adolescent hazardous alcohol consumption should also be aimed at tackling parents’ attitudes towards and consumption of alcohol.

## Background

Alcohol plays a complex role in society. It represents an integral part of modern culture and is generally consumed for reasons of enjoyment and sociability [1]. It is causally related to over 60 different medical conditions and implicated in premature deaths every year [2]. The World Health Organisation (WHO) highlight that the European Union (E.U.) is the heaviest drinking region in the world [2]. Irish people consume higher levels of alcohol when compared to the E.U. average [3] with over half of Irish adults reporting hazardous drinking [4]. This is defined as “a pattern of alcohol consumption that increases the risk of harmful consequences for the user or others” [5].

Principally alcohol is the first harmful substance misused in adolescence [6–10]. Adolescents represent a unique subsection of society, vulnerable to the harms associated with alcohol use [10–12]. Excessive alcohol use among adolescents is associated with increased drug [12] sexual health [11] and mental health issues [13, 14]. It also impacts on brain development and academic performance [15]. Young European adults report being drunk more often than their counter parts world-wide. This equates to 39 % of European students reporting being drunk in the last 30 days [16]. The rate of heavy episodic drinking by adolescents in Ireland is above the European average [16, 14]. Recent research has signalled that adolescents who drink alcohol before the age of 15 are ‘four times more likely to develop alcohol dependency than those who wait until they are 21’ [17].

A number of factors influence the onset of adolescent alcohol consumption including peer pressure, societal factors and parental influences [18–20].

1. Peer: Previous research has noted the influence of peers on adolescent consumption and other risky behaviours such as substance use during adolescence and early adulthood [20–24].
2. Culture: Alcohol consumption has it’s foundations in ancestral European culture [18]. European adolescents drink more often and at higher levels than any of their counterparts worldwide, 57 % of European adolescents reported having a drink in the last 30 days [16, 25, 26]. The alcohol industry promotes a pro-alcohol lifestyle using social-networking systems that are extensively used by adolescents. Evidence-based harm reduction policies are not widely followed [27].
3. Parent: Research has noted the impact of the parent in the formative years of an adolescent’s life. Parent alcohol consumption can be regarded by the child as acceptable behaviour to emulate [8, 28, 29]. A recent systematic review of relevant studies reports on a number of parenting strategies aimed at reducing adolescent alcohol consumption [30]. Results indicate that it is risky for parents to allow children to drink during early adolescence [17]. The risk of dependence is increased by a family history of alcohol problems [31]. It’s been reported that fathers who report heavy drinking and early onset of alcohol use is predictive of harmful alcohol use in their children [32].

However, the most recent review on the subject of parent child relationship highlighted a lack of nation specific research of this culturally specific phenomenon [33]. Furthermore, a lack of European research was noted. Thus, the aim of this current research is to investigate the relationship between parental attitude and consumption of alcohol and adolescent alcohol consumption in Southern Ireland.

## Methods

### Survey design

A cross-sectional survey was undertaken. This involved distributing a questionnaire to a sample of second-level students in the Kanturk-Mallow local electorate area (Southern Ireland). The survey was carried out during the academic year 2014–15.

### Sampling

The sample consisted of students in their final 2 years of second level education from a local electorate area in Southern Ireland, which has an estimated general population of 28,404 and a total secondary school population of 2119 across 6 year groups. According to the 2011 national census this area has a breadth of socioeconomic classes and incomes [34]. All eight secondary schools in the locality were included yielding a sample population of 982 students in their penultimate and final year.

These 982 students were asked to bring an envelope home to their parents containing a consent form and two parent surveys. Parents completed their survey and returned it in the sealed envelope provided. The consent forms were separated from the parent questionnaires on collection. This consent form outlined the aim of the study, the nature of the survey and provided detail on who was organising the research. Furthermore, it highlighted the voluntary nature of the research and an individual’s right to discontinue at any point. The front page of the survey included information on the aim, the nature of participation, who the researchers were and the benefits of completing the questionnaire. Parents consented both for themselves and their child. The child also signed the consent form in the presence of their parent or guardian. Students were surveyed in class. Prior to completion students were again advised of the aim of the study and the voluntary nature of participation. The surveys were confidential; each parent and pupil had a corresponding identification number on their survey to link results. The final page of both parent and pupil questionnaires provided information on individuals and organisations to contact in case of distress or upset as a result of taking the survey. Data collection occurred over a 2 week period during November 2014.

### Measurement

Topics in both parent and pupil surveys included alcohol use, self-reported height and weight, smoking status, mental health and well-being. The study utilised previously validated questions. Consumption questions were attained from the Alcohol Use Disorders Identification Test for Consumption (AUDIT-C) [35]. AUDIT-C takes the first three questions of the AUDIT questionnaire which focus on frequency and volume of alcohol consumption. The guidelines on low-risk alcohol consumption are lower in adolescents than those for parents, reflecting their increased vulnerability to alcohol-related harm. Based on previous national and international research, hazardous drinkers were defined as an AUDIT-C score of 5 or more amongst parents [4] and 4 or more amongst adolescents [35]. Parental attitude to alcohol questions were included in the parent survey [36, 37]. Questions on adverse consequences described in CLAN were utilised in the adolescent survey [38]. Personal wellbeing was measured using the Personal Wellbeing Index (PWI) in both adolescents [39] and parents [40]. Parental attitude questions were taken from Smyth et al. and Research New Zealand papers [36, 37]. Second-hand effect, smoking and body mass index questions were taken from the Survey of Lifestyle, Attitudes and Nutrition in Ireland (SLAN) [41] and the College Lifestyle Attitudinal National (CLAN) Survey [38]. Body mass index (BMI) was estimated from self-reported height and weight [42].

### Statistical analysis

IBM Statistical Package for Social Science (SPSS) Statistics 20 was used for statistical analysis. AUDIT-C scores were calculated according to instrument guidelines [43, 44]. Analysis included descriptive, frequency and binary logistic regression analysis. Parental attitude questions were measured on a 5-point Likert scale. To increase statistical power, strongly disagree, disagree and neutral were combined as were strongly agree and agree. Hazardous alcohol consumption of the adolescent, as described using the AUDIT-C, was the dependent variable. A stepwise multivariate logistic regression was undertaken to investigate the association between parent consumption, behaviour and demographics and adolescent alcohol consumption. The first model investigated the association between adolescent alcohol consumption and parental sociodemographic factors. The second model included parental attitude. The final model included consumption, attitude and socio-demographic information.

## Results

Three hundred and sixty students completed the questionnaire yielding a response rate of 37 % based on the number of students registered to those particular school years in the local-electorate area. Five hundred and forty-two corresponding questionnaires were retrieved from the participating students’ parents. Pupil and parent data were linked using a matching unique identification number.

### Adolescent

The median age of adolescents was 17 years. 55.8 % were female. Over one-third of adolescents reported being hazardous drinkers (34.2 %), more males (39.6 %; 95 % CI 31.9–47.3 %)) than females (29.9 %; 95 % CI 23.5–36.2 %)). 68.2 % of these hazardous drinking adolescents were under the age of 18. These results are shown in Table 1.

**Table 1:** Gender, age and lifestyle profiles of adolescents

	Adolescent		Median	Quartile 1	Quartile 3
Age			17	16	17
			Male N (%)	Female N (%)	Total N (%)
Adolescent	*Gender*				159 (44.2 %)
					201 (55.8 %)
	*Hazardous Drinking*	*Non-Hazardous*	96 (60.4 %)	141 (70.1 %)	237 (65.8 %)
		*Hazardous*	63 (39.6 %)	60 (29.9 %)	123 (34.2 %)
	*School Year*	*Fifth Year*	99 (62.3 %)	114 (56.7 %)	213 (59.2 %)
		*Sixth Year*	60 (37.7 %)	87 (43.3 %)	147 (40.8 %)

### Parent

More mothers (55.8 %) took part in the survey than fathers (44.2 %). Forty-seven percent of all parents surveyed were hazardous drinkers. The median age was 50 years for fathers and 48 years for mothers. Fathers were more likely to be hazardous drinkers (50 %) compared to mothers (46.8 %). These results are shown in Table 2.

**Table 2:** Gender, age and lifestyle profiles of parents

	Parent	Median	Quartile 1	Quartile 3
Age	*Father*	50	46	50
	*Mother*	48	44	48
			Father N (%)	Mother N (%)
Parent	*Gender*		268 (100 %)	339 (100 %)
	*Hazardous Drinking*	*Non-Hazardous*	134 (50 %)	179 (52.8 %)
		*Hazardous*	134 (50 %)	160 (47.2 %)
	*Education Status*	*Primary/Second level*	174 (65.2 %)	149 (44.0 %)
		*Third level*	93 (34.8 %)	190 (56.0 %)
	*Marital Status*	*Cohabiting/Married*	253 (94.4 %)	293 (86.4 %)
		*Single/Separated/Divorced/Widowed*	15 (5.6 %)	46 (13.6 %)

### Parent attitude

Over 90 % of parents disagreed with allowing their adolescent to get drunk. However, almost one-fifth of parents said they would not be worried if their adolescent consumed four pints of alcohol once a month. A large number of parents (42.8 %) agreed with permitting their adolescent to drink on special occasions and almost 18.2 % believed it would be okay for another parent to provide alcohol to their adolescent under supervision.

When fathers’ attitude toward alcohol was investigated further in univariate analysis it was found that if an adolescent is a hazardous drinker, their father is more likely to agree that it’s ‘Okay for their adolescent to get drunk sometimes’ (14.8 % vs. 2.8 %). Similarly, fathers of hazardous drinking adolescents were more likely to agree that ‘Getting drunk is part of having fun as an adolescent’ (13.5 % vs. 3.9 %) or that ‘It’s okay for pupils to drink on special occasions’ (65.2 % vs 29.8 %), when compared to adolescents that do not drink hazardously. Furthermore, if the adolescent is a hazardous drinker their father is more likely to ‘Allow another parent to supply their adolescent with alcohol’ (32.6 % vs. 9 %). These results are shown in Table 3.

**Table 3:** Univariate analysis: Association between parent attitude and adolescent alcohol consumption

Attitude to alcohol consumption		Fathers attitudes			Mothers attitudes		
		Child’s drinking pattern					
		Hazardous drinking N (%)	Non-hazardous drinking N (%)	*p*-value	Hazardous drinking N (%)	Non-hazardous drinking N (%)	*p*-value
It’s ok for my adolescent to get drunk sometimes	*SD/D/N*	76 (85.4 %)	173 (97.2 %)	<0.001	112 (94.1 %)	214 (98.6 %)	0.020
	*SA/A*	13 (14.6 %)	5 (2.8 %)		7 (5.9 %)	3 (1.4 %)	
Getting drunk is part of having fun as an adolescent	*SD/D/N*	77 (86.5 %)	171 (96.1 %)	0.004	108 (90.8 %)	212 (97.2 %)	0.009
	*SA/A*	12 (13.5 %)	7 (3.9 %)		11 (9.2 %)	6 (2.8 %)	
Adolescents should not drink at all	*SD/D/N*	42 (47.7 %)	72 (40.4 %)	0.259	63 (52.5 %)	80 (36.7 %)	0.005
	*SA/A*	46 (52.3 %)	106 (59.6 %)		57 (47.5 %)	138 (63.3 %)	
I set a good example for my adolescent through my own drinking	*SD/D/N*	26 (29.2 %)	42 (23.6 %)	0.321	18 (15.1 %)	34 (15.6 %)	0.909
	*SA/A*	63 (70.8 %)	136 (76.4 %)		101 (84.9 %)	184 (84.4 %)	
I teach my adolescent to drink responsibly in a home environment	*SD/D/N*	40 (44.9 %)	99 (55.6 %)	0.1	44 (36.7 %)	116 (53.2 %)	0.004
	*SA/A*	49 (55.1 %)	79 (44.4 %)		76 (63.3 %)	102 (46.8 %)	
It’s okay for adolescents to drink on special occasions	*SD/D/N*	31 (34.8 %)	125 (70.2 %)	<0.001	45 (37.5 %)	146 (67 %)	<0.001
	*SA/A*	58 (65.2 %)	53 (29.8 %)		75 (62.5 %)	72 (33 %)	
It would be ok for another parent to provide my adolescent with alcohol	*SD/D/N*	60 (67.4 %)	161 (91 %)	<0.001	80 (67.8 %)	191 (88 %)	<0.001
	*SA/A*	29 (32.6 %)	16 (9 %)		38 (32.2 %)	26 (12 %)	
It’s a good idea to introduce alcohol in the home	*SD/D/N*	58 (65.2 %)	129 (72.9 %)	0.194	72 (61 %)	155 (71.8 %)	0.044
	*SA/A*	31 (34.8 %)	48 (27.1 %)		46 (39 %)	61 (28.2 %)	
I wouldn’t be concerned if my adolescent drank 4 pints once a month	*SD/D/N*	67 (76.1 %)	155 (87.6 %)	0.017	85 (72.6 %)	192 (88.9 %)	<0.001
	*SA/A*	21 (23.9 %)	22 (12.4 %)		32 (27.4 %)	24 (11.1 %)	

*SA/A includes *Agree/Strongly agree*

*SD/D/N includes *Strongly disagree/disagree/neutral*

The mother of an adolescent reporting hazardous drinking is more likely to agree that “it’s okay for their adolescent to get drunk sometimes” (5.9 % vs. 1.4 %) or that “getting drunk is part of having fun as an adolescent” (9.2 % vs. 2.8 %). Furthermore, 47.5 % of mothers of hazardous drinking teenagers agreed that adolescents should not drink at all compared to 63.3 % of mothers with non-hazardous drinking teenagers. Mothers who felt it was ok for their child to drink on special occasions were more likely to have hazardous consuming teenagers (62.5 %). These mothers were more likely to agree with introducing alcohol in the home (39 %) and not be concerned if there adolescent “drank four pints once a month” (27.4 %). These results are shown in Table 3.

### Multivariate analysis

A stepwise multivariate logistic regression was undertaken to investigate the association between parent consumption, behaviour and demographics on adolescent alcohol consumption. Two hundred twenty-one individuals are included in the final, fully adjusted model. The fully adjusted model reports that adolescents whose fathers reported hazardous alcohol consumption were almost three times as likely to report hazardous alcohol consumption themselves (O.R = 2.90, 95 % CI: 1.32–6.35). Parental attitude toward drinking on special occasions was also positively associated with adolescent hazardous alcohol consumption. The father permitting drinking on special occasions results in adolescents being over four times more likely to report hazardous alcohol consumption (O.R = 4.60, 95 % CI: 1.19–11.18). This is over three times more likely if mothers report the same attitude (O.R = 3.79 95 % CI: 1.31–10.94). These results are shown in Table 4.

**Table 4:** Multivariate logistic regression of the association between parental factors and adolescent alcohol consumption

Parent			Crude OR	95 % CI	Adjusted OR:	95 % CI	Adjusted OR	95 % CI
Father	*Education Status*	*Third level*	0.78	0.46–1.41	1.30	0.62–2.72	1.16	0.55–2.49
	*Marital Status*	*Cohabiting/Married*	0.38	0.03–4.47	0.61	0.02–17.98	0.29	0.01–10.30
	*Age*	*Age*	0.96	0.89–1.03	0.93	0.54–1.02	0.94	0.86–1.03
Mother	*Education Status*	*Third level*	1.17	0.66–2.07	1.35	0.65–2.79	1.64	0.76–3.54
	*Marital Status*	*Cohabiting/Married*	1.94	0.15–25.30	1.20	0.04–34.35	2.40	0.07–82.94
	*Age*	*Age*	0.99	0.92–1.06	0.98	0.89–1.07	0.97	0.89–1.07
Father	*It’s ok for my adolescent to get drunk sometimes*	*SA/A*			5.39	1.21–23.99	5.03	1.13–22.40
	*Getting drunk is part of having fun as an adolescent*	*SA/A*			1.07	0.22–5.07	1.09	0.23–5.15
	*Adolescents should not drink at all*	*SA/A*			2.32	1.00–5.41	2.22	0.93–5.33
	*I set a good example for my adolescent through my own drinking*	*SA/A*			0.45	0.19–1.03	0.55	0.23–1.32
	*I teach my adolescent to drink responsibly in a home environment*	*SA/A*			1.24	0.55–2.83	1.23	0.52–2.88
	*It’s okay for adolescents to drink on special occasions*	*SA/A*			**4.88**	**2.05–11.57**	**4.60**	**1.19–11.18**
	*It would be ok for another parent to provide my adolescent with alcohol*	*SA/A*			1.66	0.52–5.24	1.56	0.48–5.11
	*It’s a good idea to introduce alcohol in the home*	*SA/A*			0.63	0.26–1.52	0.67	0.27–1.65
	*I wouldn’t be concerned if my adolescent drank 4 pints once a month*	*SA/A*			1.52	0.60–3.84	1.17	0.44–3.09
Mother	*It’s ok for my adolescent to get drunk sometimes*	*SA/A*			0.85	0.07–11.17	0.83	0.05–12.82
	*Getting drunk is part of having fun as an adolescent*	*SA/A*			0.50	0.08–3.10	0.70	0.10–4.72
	*Adolescents should not drink at all*	*SA/A*			0.86	0.37–1.99	1.10	0.46–2.62
	*I set a good example for my adolescent through my own drinking*	*SA/A*			2.19	0.72–6.62	2.22	0.71–6.94
	*I teach my adolescent to drink responsibly in a home environment*	*SA/A*			1.18	0.48–2.94	1.13	0.44–2.88
	*It’s okay for adolescents to drink on special occasions*	*SA/A*			**3.25**	**1.16–9.15**	**3.79**	**1.31–10.94**
	*It would be ok for another parent to provide my adolescent with alcohol*	*SA/A*			0.97	0.28–3.41	0.98	0.27–3.57
	*It’s a good idea to introduce alcohol in the home*	*SA/A*			0.42	0.16–1.10	0.47	0.17–1.25
	*I wouldn’t be concerned if my adolescent drank 4 pints once a month*	*SA/A*			1.46	0.48–4.51	1.43	0.44–4.65
Father	*Fathers Hazardous Drinking*	*Hazardous*					**2.90**	**1.32–6.35**
Mother	*Mothers Hazardous Drinking*	*Hazardous*					0.74	0.34–1.61

*Odds Ratios in bold are significant at *p* < 0.05

*SA/A includes Agree/Strongly agree

## Discussion

Previous research highlights the role of the parent in terms of their attitude towards gender roles, sexual behaviour and substance use [45]. Hazardous drinking by the father was closely related to hazardous drinking by the adolescent, supporting recent research findings [19]. It is noteworthy that no such effect is observed with the drinking pattern of the mother. However, mothers’ attitudes towards alcohol is associated with adolescent consumption. Introducing alcohol at home on special occasions may be causing increased levels of consumption amongst adolescents [46]. A more authoritative parenting style may have a protective effect as previously suggested [47]. As previously highlighted, a joint parenting approach is important when confronting the issue of adolescent alcohol consumption [48]. A recent Irish study found similar attitudes amongst parents, however no link with adolescent drinking patterns was investigated [36]. Internationally, Bellis et al. found that adolescents who attained alcohol from parents experienced less alcohol-related harm, this study found that mothers introducing alcohol in a controlled home environment to have a protective effect [49]. Although these results are noted in the current research, policy reviews have noted the negative impact of this behaviour [50]. A larger sampling frame is required to investigate this curious finding.

### Policy recommendations

The findings advocate the need for further public policy. In tackling alcohol consumption a variety of policy measures are required. Previous research has noted the importance of a minimum age for consumption. Exposure to alcohol in adolescence has detrimental effects on brain development and intellectual capabilities while it also increases the likelihood for later alcohol dependence [51, 52]. However, the current study notes that the majority of hazardous adolescent drinkers (68.2 %) were under the legal age of consumption. Therefore there is an urgent need to enhance our understanding of how adolescents acquire alcohol alongside improving enforcement of the law regarding under age alcohol sales. Moreover, previous research has noted the importance of restricting alcohol advertising and marketing which is aimed directly at adolescents [53–55]. The promotion of alcohol in areas such as sport, social media and music is increasingly prevalent [55]. Well-established evidence shows alcohol marketing increases the likelihood that adolescents will start to drink or will drink more if they are already using alcohol [54]. The evidence surrounding minimum unit pricing highlights its protective effects [56]. In addition, introduction of a parent programme to equip parents with the development of a culture of nurturing behaviours, establishing boundaries, and parental monitoring has shown promising results [48].

### Strengths and weaknesses

This study had a number of strengths and weaknesses. The strengths of this study were that an electorate area in Southern Ireland was sampled and this is unlikely to differ significantly from the general population. Furthermore, every student in the final two years of second level education within the electorate area were invited to participate. The proportions of male and female participants was broadly similar to national figures where males represent 48 % of the population compared to 52 % of females [57]. In addition, age demographics were similar to national statistics [57]. Finally, the proportion of numbers who participated in the research across the penultimate and final years were broadly similar to national levels [57]. Moreover, our findings regarding parental and adolescent hazardous drinking levels were similar to recent national research [1, 58]. Both parent and adolescent surveys utilised previously validated questions from international studies. This study included two parent questionnaires so that both the influence of the mother and the father could be recorded.

However, a low response rate (37 %) was attained. Selection bias cannot be ruled out as no information on those who chose not to complete the survey is available. Furthermore, response rates for parents are unavailable due to the fact that population size information is unavailable. In addition, due to low response numbers particular characteristics could not be investigated due to infrequency of reporting i.e. single mothers, education status etc. It is possible that the survey may have produced socially desirable responses, certain results such as BMI were different from similar previous studies [41, 59–61]. However the results of participants were confidential. Finally, as parent and adolescent alcohol consumption were measured simultaneously, reverse causation cannot be dismissed [62].

## Conclusion

Over one-third of adolescents were hazardous drinkers, and, of these, the majority were under the legal age of consumption. In particular, the influencing nature of both the father and the mother in terms of behaviour and attitude is noteworthy. In future, we recommend that any action plan to tackle adolescent drinking behaviours should also be aimed at tackling parent’s attitudes towards and consumption of alcohol. This research notes that the majority of hazardous adolescent drinkers (68.2 %) were under the legal age of consumption, hence an enhanced understanding of how adolescents acquire alcohol alongside improved enforcement of the law regarding under age adolescents buying alcohol is required. Furthermore, nation specific longitudinal research is required to investigate the impact of parental alcohol consumption, attitude and parenting style on adolescent alcohol consumption.

## Abbreviations

AUDIT-C: Alcohol use disorders identification test for consumption
BMI: Body mass index
CLAN: College lifestyle attitudinal national (Survey)
EU: European Union
PWI: Personal wellbeing index
SLAN: Survey of lifestyle, attitudes and nutrition (in Ireland)
SPSS: Statistical package for social science
WHO: World Health Organization
